# Weight change patterns across adulthood are associated with the risk of osteoarthritis: a population-based study

**DOI:** 10.1007/s40520-024-02792-w

**Published:** 2024-06-27

**Authors:** Aiyong Cui, Jun Zhang, Hongli Deng, Xing Wei, Yan Zhuang, Hu Wang

**Affiliations:** Department of Orthopaedics, Honghui Hospital, Xi’an Jiao Tong University, Xi’an, 710000 China

**Keywords:** Osteoarthritis, Weight change, Obesity, Adults, NHANE

## Abstract

**Background:**

Body weight has been recognized as a driving factor of osteoarthritis. Few studies had investigated the association between weight status across adulthood and risk of osteoarthritis (OA). This study investigates the association of weight change patterns across adulthood (lasting at least 25 years) with the risk of OA from the National Health and Nutrition Examination Survey (NHANES) 2013–2018.

**Methods:**

The study assessed the relationship between weight change across adulthood and OA in 7392 individuals aged > 50 spanning a minimum of 25 years. Multivariate linear regression analyses were utilized to detect the association between weight change patterns and self-reported OA. Restricted cubic splines (RCS) were used to examine the nonlinear relationship between absolute weight change and OA risk.

**Results:**

From 10 years ago to survey, the risk of OA was 1.34-fold (95% CI 1.07–1.68) in people changed from obese to non-obese, 1.61-fold (95% CI 1.29–2.00) in people change from non-obese to obese, and 1.82-fold (95% CI 1.49–2.22) in stable obese people compared with people who were at stable normal weight. Similar patterns were also observed at age 25 years to baseline and age 25 years to 10 years before the baseline. The dose–response association of RCS found a U-shaped relationship between absolute weight change and OA risk.

**Conclusions:**

The study suggests that weight patterns across adulthood are associated with the risk of OA. These findings stressed important to maintain a normal weight throughout adulthood, especially to prevent ignored weight gain in early adulthood to reduce OA risk later.

**Supplementary Information:**

The online version contains supplementary material available at 10.1007/s40520-024-02792-w.

## Introduction

Osteoarthritis (OA) is a progressive degenerative joint disease, mainly presented by articular cartilage deterioration and leading to high disability in daily activities and reduced life quality [1]. It is reported that one-third of the population over 65 years old is affected by OA, and over 500 million individuals are currently suffering from OA worldwide [1]. As a result of the global population aging and the obesity epidemic, the prevalence of OA has been increasing in recent years, placing a significant health and economic burden on society [2]. Since there is no effective cure for advanced OA except total joint arthroplasty, the treatment strategy for osteoarthritis has shifted to prevention at an early stage.

Body weight has been long regarded as a prime and possibly avoidable risk factor for OA, with significant implications for the occurrence, progression, and severity of the disease. Several epidemiologic studies have shown a strong association between overweight and OA. In a cohort study involving 1,764,061 subjects, Reyes et al. [3] found that overweight, class I obese, and class II obese individuals had a 2.0, 3.1-, and 4.7 times risk of knee OA, respectively, compared to normal-weight individuals. Excess weight not only leads to increased loads on weight-bearing joints but also to misalignment and poor lines of force in the lower extremities, which can lead to joint degeneration [4, 5]. Furthermore, excess body weight can lead to an inflammatory state in chondrocytes through FAK, JNK, ERK, NF-κB, and p38 signaling, which enhances the expression of inflammatory mediators [6, 7]. In a study involving 640 participants from the Osteoarthritis Initiative, Gersing et al. [8] explored the relationship between weight loss and cartilage changes over 48 months, and they found that cartilage degradation was significantly lower in participants who lost weight over 48 months [8].

However, most studies measured body weight at a one-time point or short-term and ignored the dynamic changes in weight across a long term. People who have just gained weight may have a different osteoarthritis risk compared to people who have been obese for years. Recently, long-term weight change across adulthood has been reported to be a key factor for cardiovascular disease, osteoporosis, diabetes, non-alcoholic fatty liver disease, and all-cause mortality [9–12]. Thus, this study aimed to investigate the association between long-term weight status across adulthood and the risk of osteoarthritis based on the National Health and Nutrition Examination Survey (NHANES) 2013–2018.

## Methods

### Study design

**Fig. 1:**
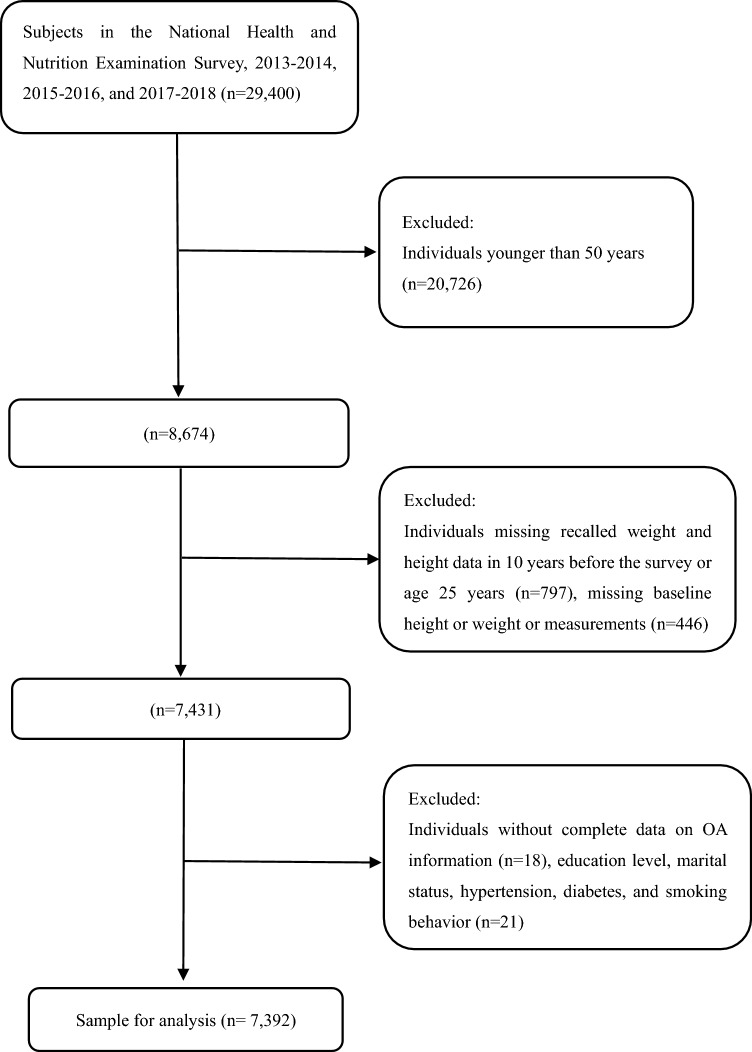
Flowchart of participants selection

The NHANES is a national nutrition and health survey on the U.S. population that collects and publicly releases data biennially. Participants or their guardians signed informed consent forms for the NHANES programs. We combined the latest NHANES data from 2013–2014, 2015–2016, and 2017–2018. Considering that osteoarthritis usually occurs in older adults, we enrolled subjects aged 50 years or older. Of 29,400 individuals from NHANES 2013–2018, 20,726 participants younger than 50 were excluded. Then, 797 individuals were excluded since missing recalled height and weight data in or age 25 years and 10 years before the survey. 446 participants were excluded because of the missing baseline height or weight measurements, 18 individuals were excluded due to missing OA information, and 21 individuals were excluded due to missing information on education level, marital status, hypertension, diabetes, and smoking behavior. Finally, 7392 individuals were enrolled in this study. The participant selection flowchart was displayed in Fig. 1.

### Osteoarthritis assessment

OA status was evaluated through a questionnaire at baseline. All participants were questioned: ‘Has a doctor or other health professional ever told you that you have arthritis?’ If participants answered yes, they were then asked, “What type of arthritis?” Those who answered yes were classified as the OA group, while those who answered otherwise were classified as the non-OA group. Study shows high concordance between OA identified by self-report and clinically diagnosed OA [13].

### Weight change assessment

Weight and height data at 10 years before the baseline of 2013–2018 and at age 25 years were recalled. Baseline height and weight were collected during the mobile physical examination. Body mass index (BMI) at 10 years before the baseline (BMI_10_), age 25 years (BMI_25_), and baseline survey (BMI_baseline_) were calculated as weight (kilograms) divided by height (meters) squared. BMI was further divided into three groups, including normal weight (< 25.0), overweight (25.0–29.9), and obesity (≥ 30.0) [14, 15]. We created weight change patterns for three intervals: BMI_10prior_ to BMI_baseline_, BMI_25_ to BMI_baseline,_ and BMI_25_ to BMI_10prior_. Then, we defined five weight change patterns for each time interval as in the previous study [9] (Supplementary 1). Stable normal pattern (BMI < 25.0 on both occasions), maximum overweight (BMI ranged from 25.0–29.9 on one occasion, < 30.0 on another), obese to non-obese (BMI ≥ 30.0 at a young age, < 30.0 later), non-obese to obese (< 30.0 at a young age, ≥ 30.0 later), and stable obesity (≥ 30.0 on both occasions). To explore the association between absolute weight change and self-reported OA, we categorized the absolute weight change into five groups: − 2.5 kg ≤ weight change ≤ 2.5 kg (control group), weight loss > 2.5 kg, 2.5 kg < weight gain < 10 kg, 10 kg ≤ weight gain ≤ 20 kg, and weight gain ≥ 20.0 kg [16].

### Covariates measurements

Covariates were chosen based on the published studies to eliminate potential effects on the final results [17, 18]. Sociodemographic characteristics such as race/ethnicity, age, sex, poverty income ratio (PIR), marital status, and education level was collected through self-reported questionnaires. Race/ethnicity was divided into five groups (Non-Hispanic White, Non-Hispanic Black, Mexican American, Other Hispanic, and Other Race). PIR was measured by dividing total household income by the poverty line. We classified it into three groups (< 1.3, 1.3–1.5, and ≥ 3.5). Education level was classified into three groups (College degree or above, High school graduate, and Under high school). Marital status was also categorized into three groups (Married/cohabiting, Widowed/divorced/separated, and Never married). Smoking behavior, diabetes and hypertension were determined by questionnaires: Smoke at least 100 cigarettes in life (Yes, No)? Doctor told you have diabetes (Yes, No)? Doctor told you have hypertension (Yes, No)? Physical activity was determined by questionnaire: In a typical week, on how many days do you do moderate-intensity sports, fitness or recreational activities? We divided the answers into two groups (≤ 3 days, ≥ 4 days).

### Statistical analysis

Mean ± standard deviation and percentages were used to represent continuous and categorical variables. Pearson correlation coefficients were calculated between weight change in the three intervals and BMI at the three time points. To compare differences between weight change pattern groups at three intervals, weight-adjusted analysis of variance and Rao-Scott χ2 test were employed for numerical and categorical characteristics. In primary analysis, logistic regression models were performed to determine the association of weight change patterns with the risk of osteoarthritis in the three intervals. The subgroup analyses (Gender (male, female), Age (< 65, ≥ 65), PIR (< 1.3, 1.3–3.5, > 3.5)) were performed to test the heterogeneity and interaction effects. From age 25 years to 10 years before baseline approximates the period from young to middle age. From 10 years before the survey to baseline approximates the period from middle to late adulthood. From age 25 to baseline approximates the period from weight change throughout adulthood. We also explored the relationship between absolute weight change groups or weight change per 5 kg and OA risk in the above three intervals. We first built an unadjusted model (Model 1). Then, Model 2 was created by adjusting race/ethnicity, age, and sex. Finally, Model 3 was created by adjusting all variables of race/ethnicity, age, sex, PIR, education level, marital status, smoking behavior, diabetes, hypertension, and physical activity. Then, logistic regression models with restricted cubic splines (RCS) of three knots (5th, 50th, and 95th percentiles) were used to examine the nonlinear association between absolute weight change and osteoarthritis risk. All analyses were performed by R software (4.3.1), with *P* values < 0.05 regarded as statistically significant.

## Results

### Baseline characteristics

The Pearson correlation coefficients between BMI_25_, BMI_10prior_, and BMI_baseline_ varied from 0.43 to 0.71 (Supplementary 2). A sample of 7392 subjects were recruited in our analyses, of which 49.53% were males and 50.47% were females, with a mean age of 64.79 ± 9.34 years. 1541 (20.85%) individuals were reported to have osteoarthritis at baseline. The average BMI at age 25 years, 10 years before the baseline, and baseline at NHANES 2013–2018 were 23.37 ± 4.58, 28.82 ± 6.63, and 29.56 ± 6.74 kg/m^2^. From 10 years before the survey to baseline, 1339 (18.1%) individuals were stable non-obese, 2058 (27.8%) individuals were maximum overweight, 988 (13.4%) individuals changed from obese to non-obese, 1074 (14.5%) individuals change from non-obesity to obesity, and 1933 (26.1%) individuals were stable obesity. Compared to those in the stable-normal group, those in the other groups were more likely to be older, men, Mexican American, Other Hispanic, Non-Hispanic Black, less educated, never married, poor, and smokers. They are more likely to report having OA, diabetes, hypertension, and have less physical exercise (Table 1). Similar characteristics and trends of participants in the other two intervals were also observed (Supplementary 3 and 4).

**Table 1:** Baseline characteristics of study participants in NHANES 2013–2018 based on weight change patterns from 10 years ago to baseline

Characteristics	Total	Weight change patterns from 10 years ago to baseline					P value
		Stable normal weight	Maximum overweight	Obesity to non-obesity	Non-obesity to obesity	Stable obesity	
N	7392	1339 (18.1%)	2058 (27.8%)	988 (13.4%)	1074 (14.5%)	1933 (26.1%)	< 0.001
Age, years, mean ± SE	64.79 ± 9.34	64.15 ± 9.55	65.15 ± 9.49	67.61 ± 9.35	62.39 ± 8.73	64.75 ± 8.92	< 0.001
Gender, n (%)							< 0.001
Men	3661 (49.53%)	606 (45.26%)	1137 (55.25%)	589 (59.62%)	414 (38.55%)	915 (47.34%)	
Women	3731 (50.47%)	733 (54.74%)	921 (44.75%)	399 (40.38%)	660 (61.45%)	1018 (52.66%)	
Race/ethnicity, n (%)							< 0.001
Mexican American	874 (11.82%)	72 (5.38%)	252 (12.24%)	135 (13.66%)	133 (12.38%)	282 (14.59%)	
Other Hispanic	758 (10.25%)	103 (7.69%)	237 (11.52%)	95 (9.62%)	144 (13.41%)	179 (9.26%)	
Non-Hispanic White	3138 (42.45%)	562 (41.97%)	872 (42.37%)	426 (43.12%)	416 (38.73%)	862 (44.59%)	
Non-Hispanic Black	1632 (22.08%)	227 (16.95%)	391 (19.00%)	211 (21.36%)	301 (28.03%)	502 (25.97%)	
Other race	990 (13.39%)	375 (28.01%)	306 (14.87%)	121 (12.25%)	80 (7.45%)	108 (5.59%)	
Education level, n (%)							< 0.001
Under high school	1631 (22.06%)	263 (19.64%)	438 (21.28%)	275 (27.83%)	228 (21.23%)	427 (22.09%)	
High school graduate	1772 (23.97%)	304 (22.70%)	489 (23.76%)	228 (23.08%)	287 (26.72%)	464 (24.00%)	
College degree or above	3989 (53.96%)	772 (57.65%)	1131 (54.96%)	485 (49.09%)	559 (52.05%)	1042 (53.91%)	
Marital status, n (%)							< 0.001
Married/cohabiting	4418 (59.77%)	810 (60.49%)	1315 (63.90%)	564 (57.09%)	613 (57.08%)	1116 (57.73%)	
Widowed/divorced/separated	2432 (32.90%)	442 (33.01%)	609 (29.59%)	356 (36.03%)	367 (34.17%)	658 (34.04%)	
Never married	542 (7.33%	87 (6.50%)	134 (6.51%)	68 (6.88%)	94 (8.75%)	159 (8.23%)	
PIR, n (%)							< 0.001
< 1.3	1867 (25.26%)	334 (24.94%)	470 (22.84%)	286 (28.95%)	290 (27.00%)	487 (25.19%)	
1.3–3.5	3388 (45.83%)	577 (43.09%)	924 (44.90%)	472 (47.77%)	491 (45.72%)	924 (47.80%)	
> 3.5	2137 (28.91%)	428 (31.96%)	664 (32.26%)	230 (23.28%)	293 (27.28%)	522 (27.00%)	
PIR, mean ± SE	2.62 ± 1.53	2.74 ± 1.58	2.75 ± 1.55	2.42 ± 1.46	2.54 ± 1.52	2.56 ± 1.51	< 0.001
Smoke at least 100 cigarettes in life, n (%)							0.003
Yes	3668 (49.62%)	632 (47.20%)	1012 (49.17%)	543 (54.96%)	544 (50.65%)	937 (48.47%)	
No	3724 (50.38%)	707 (52.80%)	1046 (50.83%)	445 (45.04%)	530 (49.35%)	996 (51.53%)	
Self-reported hypertension, n (%)							< 0.001
Yes	4124 (55.79%)	488 (36.45%)	1064 (51.70%)	580 (58.70%)	657 (61.17%)	1335 (69.06%)	
No	3268 (44.21%)	851 (63.55%)	994 (48.30%)	408 (41.30%)	417 (38.83%)	598 (30.94%)	
Self-reported diabetes, n (%)							< 0.001
Yes	1682 (22.75%)	119 (8.89%)	330 (16.03%)	302 (30.57%)	209 (19.46%)	722 (37.35%)	
No	5394 (72.97%)	1179 (88.05%)	1657 (80.52%)	638 (64.57%)	809 (75.33%)	1111 (57.48%)	
Borderline	316 (4.27%)	41 (3.06%)	71 (3.45%)	48 (4.86%)	56 (5.21%)	100 (5.17%)	
Day moderate recreational activities, n (%)							< 0.001
< = 3 day	1579 (55.33%)	296 (22.11%)	486 (23.62%)	181 (18.32%)	240 (22.35%)	376 (19.45%)	
> = 4 day	1275 (44.67%)	294 (21.96%)	362 (17.59%)	188 (19.03%)	182 (16.95%)	249 (12.88%)	
No reported	4538 (61.39%)	749 (55.94%)	1210 (58.79%)	619 (62.65%)	652 (60.71%)	1308 (67.67%)	
Self-reported osteoarthritis, n (%)	1541 (20.85%)	211 (15.76%)	361 (17.54%)	202 (20.45%)	248 (23.09%)	519 (26.85%)	< 0.001
BMI_25_, kg/m^2^, mean ± SE	23.37 ± 4.58	20.66 ± 2.43	22.29 ± 2.94	23.96 ± 4.76	22.87 ± 3.66	26.36 ± 5.68	< 0.001
BMI_10prior_, kg/m^2^, mean ± SE	28.82 ± 6.63	22.15 ± 1.89	25.99 ± 2.26	30.49 ± 4.30	27.02 ± 2.35	36.60 ± 6.66	< 0.001
BMI_baseline_ kg/m^2^, mean ± SE	29.56 ± 6.74	21.93 ± 2.09	27.29 ± 1.37	25.67 ± 2.77	33.37 ± 3.55	37.12 ± 6.25	< 0.001

Mean ± SD for continuous variables: the P value was calculated by the weight-adjusted analysis of variance. (%) for categorical variables: the P value was calculated by the weighted Rao-Scott χ^2^ test

*BMI* body mass index, *PIR* poverty income ratio

### Association between weight change pattern and self-reported osteoarthritis

The relationship between weight change patterns and self-reported osteoarthritis was presented in Table 2. From 10 years before baseline to baseline, compared with people stay normal weight, the risk of osteoarthritis was 1.34-fold (OR:1.34, 95% CI 1.07–1.68) in people changed from obese to non-obese, 1.61-fold (OR: 1.61, 95% CI 1.29–2.00) in people changed from non-obese to obese, and 1.82-fold (OR: 1.82, 95% CI 1.49–2.22) in stable obese people. From age 25 years ago to baseline, compared with people stayed normal weight, the risk of osteoarthritis was 1.49-fold (OR: 1.49, 95% CI 1.04–2.15) in people changed from obese to non-obese, and 1.69-fold (OR: 1.69, 95% CI 1.42–2.01) in people changed from non-obese to obese, and 2.09-fold (OR: 2.09, 95% CI 1.57–2.78) in stable obese people. From age 25 to 10 years before baseline, compared with people who stayed normal weight, the risk of osteoarthritis was 1.34-fold (OR:1.34, 95% CI 1.14–1.58) in people with maximum overweight, 1.63-fold (OR: 1.63, 95% CI 1.03–2.58) in people change from obese to non-obese, 1.66-fold (OR: 1.66, 95% CI 1.40–1.97) in people changed from non-obese to obese, and 2.02-fold (OR: 2.02, 95% CI 1.55–2.64) stable obese people.

**Table 2:** Association of weight change pattern and self-reported osteoarthritis in American adults, NHANES 2013–2018

Self-reported osteoarthritis	Weight change patterns				
	Stable normal weight	Maximum overweight	Obese to non-obese	Non-obese to obese	Stable obese
From 10 years ago to baseline					
Model 1	1.00	1.14 (0.94, 1.36) 0.175	1.37 (1.11, 1.70) 0.003	1.61 (1.31, 1.97) < 0.001	1.96 (1.64, 2.35) < 0.001
Model 2	1.00	1.23 (1.01, 1.49) 0.037	1.44 (1.15, 1.80) 0.001	1.80 (1.45 2.23) < 0.001	2.13 (1.76, 2.57) < 0.001
Model 3	1.00	1.13 (0.93, 1.38) 0.221	1.34 (1.07, 1.68) 0.012	1.61 (1.29, 2.00) < 0.001	1.82 (1.49, 2.22) < 0.001
From age 25 years to baseline					
Model 1	1.00	1.17 (0.99, 1.38) 0.059	1.12 (0.79, 1.59) 0.525	1.75 (1.49, 2.05) < 0.001	1.86 (1.43, 2.42) < 0.001
Model 2	1.00	1.29 (1.08, 1.53) 0.004	1.52 (1.06, 2.18) 0.023	1.93 (1.63, 2.28) < 0.001	2.44 (1.85, 3.21) < 0.001
Model 3	1.00	1.19 (1.00, 1.41) 0.057	1.49 (1.04, 2.15) 0.032	1.69 (1.42, 2.01) < 0.001	2.09 (1.57, 2.78) < 0.001
From age 25 years to 10 years before baseline					
Model 1	1.00	1.30 (1.12, 1.52) < 0.001	1.21 (0.78, 1.87) 0.391	1.80 (1.54, 2.09) < 0.001	1.79 (1.40, 2.29) < 0.001
Model 2	1.00	1.42 (1.22, 1.67) < 0.001	1.60 (1.02, 2.51) 0.041	1.86 (1.58, 2.19) < 0.001	2.29 (1.77, 2.96) < 0.001
Model 3	1.00	1.34 (1.14, 1.58) < 0.001	1.63 (1.03, 2.58) 0.037	1.66 (1.40, 1.97) < 0.001	2.02 (1.55, 2.64) < 0.001

Model 1: no covariates were adjusted. Model 2: age, sex, and race/ethnicity were adjusted. Model 3: age, sex, race/ethnicity, PIR, education level, marital status, smoking behavior, diabetes, hypertension, and physical activity

*PIR* poverty income ratio

**Fig. 2:**
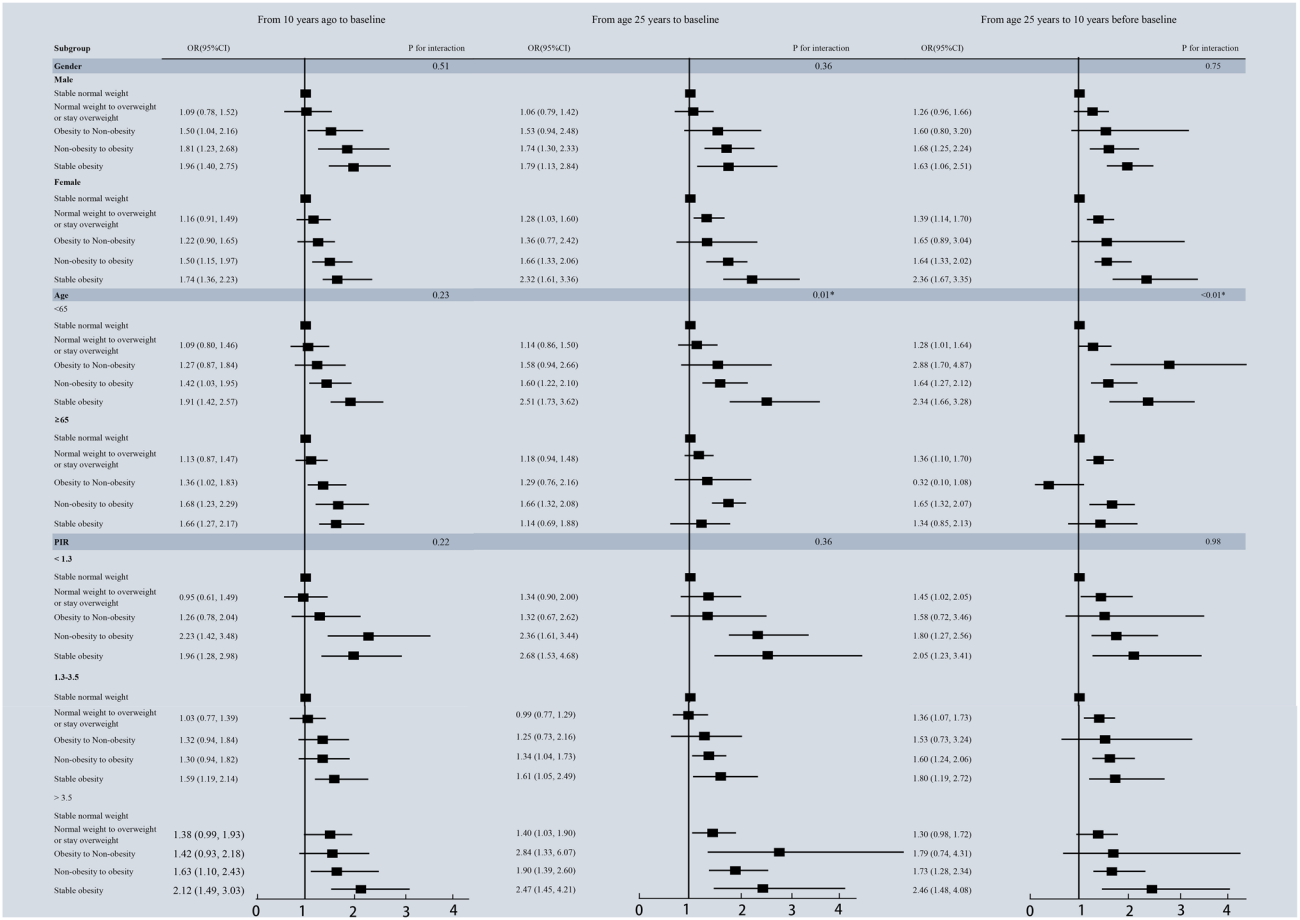
Associations between weight change patterns across adulthood and risk of osteoarthritis stratified by baseline age, sex, and, PIR in NHANES 2013–2018. Age, sex, race/ethnicity, PIR, education level, marital status, smoking behavior, diabetes, hypertension, and physical activity were adjusted. *PIR* poverty income ratio

The subgroup analyses indicated that age (< 65, ≥ 65 years) could interact with the association between weight change pattern and self-report osteoarthritis at two intervals (P for interaction < 0.05) (From age 25 to 10 years before baseline; From age 25 to baseline), as seen in Fig. 2. However, we observed no significant differences in the association between weight change patterns and self-report osteoarthritis by gender, age, and PIR categories in any other time intervals (P for interaction > 0.05).

### Association of absolute weight change and self-reported osteoarthritis

From 10 years before baseline to baseline, the multiple linear models showed that with every 5 kg increase in body weight, the risk of OA could increase by 3% (OR, 1.03; 95 CI 1.00, 1.05) (Table 3). Using the weight change within 2.5 kg as the reference, the risk for osteoarthritis in people with weight gain within 10 to 20 kg and weight gain more than 20 kg increased by 1.36-fold (OR 1.36; 95% CI 1.11–1.68) and 1.58-fold (OR, 1.58; 95% CI 1.23–2.03). From age 25 years to baseline, using the weight change within 2.5 kg as the reference, weight gain of more than 20 kg had a 1.3-fold (OR 1.30; 95% CI 1.04–1.64) higher OA risk in later years. Every 5 kg increase in body weight was associated with a 4% increase in osteoarthritis risk (OR 1.04; 95 CI 1.02, 1.06). From age 25 years to 10 years before the survey, using the weight change within 2.5 kg as the reference, weight gain of more than 20 kg (OR 1.34; 95% CI 1.11–1.62) had a higher osteoarthritis risk. Every 5 kg increase in body weight was associated with a 3% (OR 1.03; 95 CI 1.01, 1.05) increase in osteoarthritis risk in later years.

**Table 3:** Association of absolute weight change patterns and self-reported osteoarthritis in American adults, NHANES 2013–2018

Self-reported osteoarthritis	Weight change patterns					
	Linear model (per 5 kg)	Weight change within 2.5 kg	Weight loss > 2.5 kg	Weight gain > 2.5 kg and < 10.0 kg	Weight gain ≥ 10 kg and	Weight gain ≥ 20 kg
					< 20 kg	
From 10 years ago to baseline						
Model 1	1.01 (0.99, 1.04) 0.153	1	1.11 (0.94, 1.31) 0.207	0.951 (0.80, 1.13) 0.567	1.16 (0.96, 1.41) 0.134	1.27 (1.01, 1.60) 0.043
Model 2	1.03 (1.01, 1.05) 0.014	1	1.13 (0.96, 1.34) 0.141	1.05 (0.88, 1.25) 0.604	1.46 (1.19, 1.79) < 0.001	1.73 (1.36, 2.21) < 0.001
Model 3	1.03 (1.00, 1.05) 0.020	1	1.08 (0.91, 1.28) 0.368	1.00 (0.83, 1.19) 0.977	1.36 (1.11, 1.68) 0.003	1.58 (1.23, 2.03) < 0.001
From age 25 years to baseline						
Model 1	1.05 (1.04, 1.07) < 0.001	1	1.06 (0.82, 1.37) 0.645	0.86 (0.68, 1.09) 0.221	0.92 (0.74, 1.15) 0.475	1.29 (1.04, 1.60) 0.022
Model 2	1.06 (1.04, 1.08) < 0.001	1	1.05 (0.81, 1.36) 0.719	0.90 (0.71, 1.15) 0.398	0.99 (0.78, 1.24) 0.908	1.49 (1.19, 1.86) < 0.001
Model 3	1.04 (1.02, 1.06) < 0.001	1	1.09 (0.83, 1.42) 0.541	0.87 (0.68, 1.11) 0.252	0.92 (0.73, 1.16) 0.468	1.30 (1.04, 1.64) 0.023
From age 25 years to 10 years before baseline						
Model 1	1.06 (1.04, 1.08) < 0.001	1	1.23 (0.94, 1.62) 0.134	1.04 (0.86, 1.25) 0.687	1.20 (1.00, 1.45) 0.045	1.65 (1.38, 1.98) < 0.001
Model 2	1.05 (1.03, 1.07) < 0.001	1	1.24 (0.93, 1.64) 0.139	1.08 (0.89, 1.30) 0.429	1.21 (1.00, 1.46) 0.046	1.62 (1.35, 1.95) < 0.001
Model 3	1.03 (1.01, 1.05) 0.011	1	1.20 (0.90, 1.59) 0.209	0.99 (0.82, 1.20) 0.918	1.07 (0.88, 1.29) 0.496	1.34 (1.11, 1.62) 0.003

Model 1: no covariates were adjusted. Model 2: age, sex, and race/ethnicity were adjusted. Model 3: age, sex, race/ethnicity, PIR, education level, marital status, smoking behavior, diabetes, hypertension, and physical activity

*PIR* poverty income ratio

**Fig. 3:**
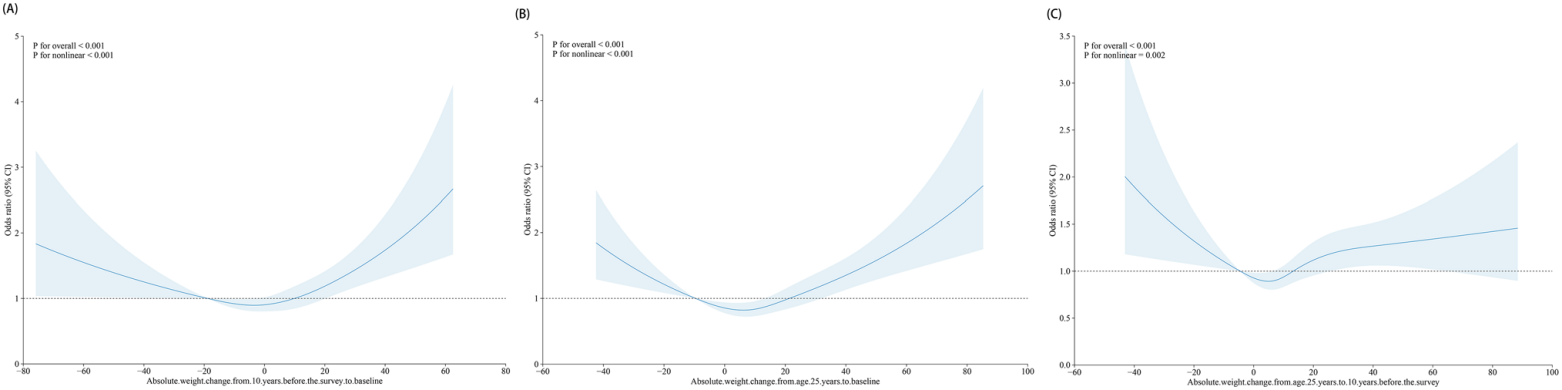
Dose–response association of the absolute weight change and risk of osteoarthritis. **A** From 10 years before baseline to baseline. **B** age 25 years to baseline. **C** From age 25 to 10 years before the baseline. Restricted cubic splines were used with three knots (5th, 50th, and 95th percentiles). Odds ratios were indicated by solid lines and 95% CI by shaded areas. Covariates included age, sex, race/ethnicity, PIR, education level, marital status, smoking behavior, diabetes, hypertension, and physical activity. *PIR* poverty income ratio

The dose–response association of RCS found a U-shaped relationship between absolute weight change and osteoarthritis risk in 10 years before baseline to baseline (P for non-linear < 0.001), age 25 years to baseline (P for non-linear < 0.001), and in age 25 years to 10 years before baseline (P for non-linear = 0.002) (Fig. 3).

## Discussion

In this study with a large population from NHANES, our study found that weight patterns across adulthood are associated with the risk of OA. People who maintain a healthy weight in early and late adulthood have a reduced risk of developing osteoarthritis. The risk of osteoarthritis was highest among those who were obese in both early and late adulthood. People who are obese in early adulthood and return to non-obese in late adulthood are also at a higher risk of osteoarthritis. These findings emphasize the critical need to maintain a normal weight throughout adulthood, especially to prevent ignored weight gain in early adulthood to reduce the risk of osteoarthritis later.

Many cohort studies have examined the relationship between body weight or obesity and osteoarthritis risk [19–21]. Generally speaking, most studies show that being overweight or obese may increase the risk of osteoarthritis. For example, in a survey that included 14,766 men and 15,191 women, Mork et al. [22] found that obesity can increase the risk of OA by 2.78 and 4.37-fold in men and women, respectively. In another study from the Mini-Finland Health Survey, Toivanen et al. [23] conducted a study with a follow-up of over 20 years (1978–80 to 2000–01). The study showed that individuals with a BMI of 25.0–29.9 kg/m^2^ and ≥ 30.0 kg/m^2^ had 1.7- and 7.0 times OA risk compared to those with BMI < 25 kg/m^2^. Weight change is a dynamic process, and several studies have explored the relationship between short-term weight change (usually several years) and OA risk. For instance, a recent longitudinal study with a 4-year follow-up suggested that individuals with four years of weight loss were related to an alleviated knee radiographic OA and pain resolution [24]. However, weight change was not associated with hip OA or total hip arthroplasty [24]. Salis et al. [25] came the same conclusion in a study with up to eight years of follow-up. They found that weight loss may not improve the progression of hip osteoarthritis in older women. The hip joint has a ball and socket anatomy with mechanical forces evenly distributed over the joint. Therefore, the hip joint is less sensitive to weight changes and obesity. However, a study from the Osteoarthritis Initiative (OAI) showed that increased body weight was positively associated with a higher risk of total knee and hip replacement [26]. Also, another study based on magnetic resonance imaging (MRI) showed that subjects who gained weight over two years had increased meniscal injuries compared with weight stabilizers [27]. Gersing et al. [8] suggested that cartilage degradation was significantly lower in participants who lost weight over 48 months. However, most of these studies have a short follow-up and may not reflect the relationship between long-term or whole-life weight change and OA occurrence. Thus, using a retrospective study spanning a minimum of 25 years, we filled in the gaps in the effect of weight change patterns throughout adulthood on the risk of OA.

Currently, worldwide clinical guidelines recommend weight loss for patients with osteoarthritis whose body mass index is in the overweight or obese range [28–32]. However, considering our results, weight loss solely in patients with osteoarthritis who are overweight or obese may not be sufficient because our study suggests that even the effects of obesity in early adulthood on osteoarthritis risk may extend into late adulthood or old age. Most of the weight gain in adults occurs during youth to middle age, but research on this critical period is still lacking [33]. Our study suggested that the risk for those who were obese at age 25 and currently non-obese was 1.39-fold compared to those who consistently maintained a normal weight. Similar to our findings, Wang et al. [20]. investigate the association between weight change from 18–21 years to middle age and risk for osteoarthritis. They found that individuals with weight gain from age 18 to 21 years to middle age had a higher risk for osteoarthritis, total hip arthroplasty (THA), and total knee arthroplasty (TKA) compared with a stable weight. However, the average investigation period in this study was only 8 years and does not reflect weight change patterns throughout adulthood. Weight change across adulthood has been recognized as an essential factor in health, including hypertension, heart failure, diabetes, non-alcoholic fatty liver disease (NAFLD), all-cause mortality, and others [9–12, 34]. In an earlier case–control study, Manninen et al. [35] explored the association between weight changes between ages 20–50 years and the risk of severe OA requiring TKA. They found that weight gain can increase the risk of knee OA. Unexpectedly, their results showed that individuals shaft from a normal weight at the age of 20 years to overweight (BMI > 25 kg/m^2^) later had a 3.15 times risk of severe OA compared with people with stable normal weight (BMI ≤ 25 kg/m^2^). In contrast, individuals who were consistently overweight had only 2.37 times the risk of OA. Their explanation for the results was that the knee could adapt to chronic overweight. However, it is worth noting that this study included only 220 cases and 415 controls, which may have reduced the reliability of the findings.

Our study emphasizes that maintaining a normal weight throughout adulthood can help avoid OA. Also, our study suggests that the effects of obesity in early adulthood on osteoarthritis risk may extend into late adulthood or old age. In early adulthood, weight gain is mainly due to the accumulation of fat mass [36]. In contrast, in late adulthood, weight gain is usually attributed to a decrease in lean body mass and an increase in fat mass [37]. The causal relationship between weight gain and increased risk of OA can be revealed in several ways. Excess weight not only increases the load on the load-bearing joints but also leads to altered lines of force and decreased joint stability due to decreased muscle strength [38, 39]. Also, in obese conditions, the pro-inflammatory state of adipose tissue macrophages (ATMs) in adipose tissue is transformed, leading to an increase in the production of pro-inflammatory cytokines, chemokines, and reactive oxygen species, which promotes the development of osteoarthritis [40, 41]. However, achieving and maintaining a normal weight can be challenging for many people. People tend to gain weight as they age [42]. Only 3.41% participants in our study change obese to no-obese from age 25 years to baseline. Thus, it is important to maintain a normal weight throughout adulthood, especially to prevent weight gain in early adulthood to reduce the risk of osteoarthritis later in life.

Our results have several advantages. First, this is the first study to explore the relationship between weight change and OA risk in a U.S. population using the most recent cycles from NHANES database, which is representative of the general U.S. population. Second, unlike other studies that explored the effect of weight change on OA over several years on osteoarthritis, our study showed the long-term impact of weight change patterns at different life stages. We introduced three intervals, from youth to middle age, from middle age to late adulthood, and throughout adulthood, which provides strong evidence on the prediction of OA risk by weight change patterns at different stages of life. Our study also has some limitations. First, we adjusted for many potential confounders, including socioeconomic status, lifestyle, and other health factors. However, most factors were collected through questionnaires and recall, which may be subject to recall bias and inaccuracies. Second, The NHANES database only records the presence or absence of OA, not the specific location. Therefore, more studies with more robust evidence are needed to explore the effects of weight change patterns on different types of OA at different life stages. Third, the diagnosis of OA is made through self-recall. Although studies have shown an 85% concordance between self-reported OA and clinically confirmed OA [43], it is still not a substitute for clinical diagnosis. Fourth, the three time points in this study can only roughly reflect the pattern of weight change in adulthood, as people in each of these groups could have weight cycled in the intervening years. Therefore, our results should be interpreted more cautiously. Furthermore, weight change is an outcome not an intervention, and future research needs to explore the effects of various interventions on OA risk. Fifth, we must acknowledge that the covariates are measured after the independent variable. This may not be rigorous, so future prospective studies are needed to validate our conclusions. In addition, there may also be unavoidable selection bias in our study. Some of the patients who had severe OA, died or were in poor health in the 2013–2018 cycle may not have been included in our study.

## Conclusions

Our findings suggest that weight patterns across adulthood are associated with the risk of osteoarthritis. The risk of osteoarthritis was highest among those who were obese in both early and late adulthood. At the same time, people who are obese in early adulthood and return to normal weight in late adulthood are also at a higher risk of osteoarthritis. These findings emphasize the importance of maintaining a normal weight throughout adulthood, especially to prevent weight gain in early adulthood to reduce the risk of osteoarthritis later in life.

### Supplementary Information

Below is the link to the electronic supplementary material.

Supplementary file1 (DOCX 12 KB)

Supplementary file2 (DOCX 16 KB)

Supplementary file3 (DOCX 24 KB)

Supplementary file4 (DOCX 24 KB)

## Data Availability

The datasets generated during and/or analysed during the current study are available in the [NHANES] repository, [https://www.cdc.gov/nchs/nhanes/].
