# Supplementary material for: Weight change patterns across adulthood are associated with the risk of osteoarthritis: a population-based study
Source: Aging Clin Exp Res. 2024 Jun 27;36(1):138. doi: 10.1007/s40520-024-02792-w (PMC11211181; doi:10.1007/s40520-024-02792-w)
Supplement: Supplementary file 1 — Supplementary file1 (DOCX 12 KB) [file 40520_2024_2792_MOESM1_ESM.docx]

| Supplementary 1. The definition of weight change patterns at three defined intervals. | | | |
| --- | --- | --- | --- |
| **From 10 years ago to baseline** |  |  |  |
| BMI at 10 years before baseline | BMI at Baseline | | |
|  | <25 kg/m^2^ | 25-29.9 kg/m^2^ | ≥30 kg/m^2^ |
| <25 kg/m2 | Stable normal weight | Maximum overweight | Non-obese to obese |
| 25-29.9 kg/m2 | Maximum overweight | Maximum overweight | Non-obese to obese |
| ≥30 kg/m2 | Obese to non-obese | Obese to non-obese | Stable obese |
| **From age 25 years to baseline** |  |  |  |
| BMI at age 25 years | BMI at Baseline | | |
|  | <25 kg/m^2^ | 25-29.9 kg/m^2^ | ≥30 kg/m^2^ |
| <25 kg/m2 | Stable normal weight | Maximum overweight | Non-obese to obese |
| 25-29.9 kg/m2 | Maximum overweight | Maximum overweight | Non-obese to obese |
| ≥30 kg/m2 | Obese to non-obese | Obese to non-obese | Stable obese |
| **From age 25 years to 10 years before baseline** |  |  |  |
| BMI at age 25 years | BMI at 10 years before baseline | | |
|  | <25 kg/m^2^ | 25-29.9 kg/m^2^ | ≥30 kg/m^2^ |
| <25 kg/m2 | Stable normal weight | Maximum overweight | Non-obese to obese |
| 25-29.9 kg/m2 | Maximum overweight | Maximum overweight | Non-obese to obese |
| ≥30 kg/m2 | Obese to non-obese | Obese to non-obese | Stable obese |

Abbreviation: BMI, body mass index.
