# Supplementary material for: Weight change patterns across adulthood are associated with the risk of osteoarthritis: a population-based study
Source: Aging Clin Exp Res. 2024 Jun 27;36(1):138. doi: 10.1007/s40520-024-02792-w (PMC11211181; doi:10.1007/s40520-024-02792-w)
Supplement: Supplementary file 2 — Supplementary file2 (DOCX 16 KB) [file 40520_2024_2792_MOESM2_ESM.docx]

| Supplementary 2: Pearson correlation coefficients for BMI at three time points and absolute weight changes during three intervals. | | | | | | | |
| --- | --- | --- | --- | --- | --- | --- | --- |
|  | BMI | | |  | Absolute weight change | | |
|  | At age 25 years | At 10 years before baseline | At baseline |  | From age 25 years to baseline | From 10 years before baseline to baseline | From age 25 years to 10 years before baseline |
| At age 25 years | 1.00 | - | - |  | - | - | - |
| At 10 years before baseline | 0.54 | 1.00 | - |  | - | - | - |
| At baseline | 0.43 | 0.71 | 1.00 |  | - | - | - |
| From age 25 years to baseline | -0.14 | 0.32 | 0.74 |  | 1.00 | - | - |
| From 10 years before baseline to baseline | -0.23 | -0.36 | 0.40 |  | 0.57 | 1.00 | - |
| From age 25 years to 10 years before baseline | -0.13 | 0.72 | 0.48 |  | 0.62 | -0.30 | 1.00 |
