# Supplementary material for: Weight change patterns across adulthood are associated with the risk of osteoarthritis: a population-based study
Source: Aging Clin Exp Res. 2024 Jun 27;36(1):138. doi: 10.1007/s40520-024-02792-w (PMC11211181; doi:10.1007/s40520-024-02792-w)
Supplement: Supplementary file 3 — Supplementary file3 (DOCX 24 KB) [file 40520_2024_2792_MOESM3_ESM.docx]

| Supplementary 3: Baseline characteristics of study participants in NHANES 2013-2018 based on weight change patterns from age 25 years to baseline. | | | | | | | |
| --- | --- | --- | --- | --- | --- | --- | --- |
| Characteristics |  | Weight change patterns from 10 years ago to baseline | | | | | P value |
|  | Total | Stable normal weight | Maximum | Obesity to | Non-obesity to obesity | Stable obesity |  |
|  |  |  | overweight | Non-obesity |  |  |  |
| **N** | 7392 | 1648 (22.29%) | 2485 (33.62%) | 252 (3.41%) | 2623 (35.48%) | 384 (5.19%) | <0.001 |
| **Age, years, mean ± SE** | 64.79 ± 9.34 | 65.19 ± 9.75 | 65.59 ± 9.47 | 64.86 ± 9.19 | 64.31 ± 9.00 | 61.16 ± 7.84 | <0.001 |
| **Gender, n (%)** |  |  |  |  |  |  | <0.001 |
| Men | 3661 (49.53%) | 772 (46.84%) | 1384 (55.69%) | 176 (69.84%) | 1146 (43.69%) | 183 (47.66%) |  |
| Women | 3731 (50.47%) | 876 (53.16%) | 1101 (44.31%) | 76 (30.16%) | 1477 (56.31%) | 201 (52.34%) |  |
| **Race/ethnicity, n (%)** |  |  |  |  |  |  | <0.001 |
| Mexican American | 874 (11.82%) | 92 (5.58%) | 329 (13.24%) | 38 (15.08%) | 360 (13.72%) | 55 (14.32%) |  |
| Other Hispanic | 758 (10.25%) | 131 (7.95%) | 286 (11.51%) | 18 (7.14%) | 289 (11.02%) | 34 (8.85%) |  |
| Non-Hispanic White | 3138 (42.45%) | 711 (43.14%) | 1061 (42.70%) | 88 (34.92%) | 1124 (42.85%) | 154 (40.10%) |  |
| Non-Hispanic Black | 1632 (22.08%) | 281 (17.05%) | 472 (18.99%) | 76 (30.16%) | 683 (26.04%) | 120 (31.25%) |  |
| Other Race | 990 (13.39%) | 433 (26.27%) | 337 (13.56%) | 32 (12.70%) | 167 (6.37%) | 21 (5.47%) |  |
| **Education level, n (%)** |  |  |  |  |  |  | <0.001 |
| Under high school | 1631 (22.06%) | 338 (20.51%) | 550 (22.13%) | 88 (34.92%) | 569 (21.69%) | 86 (22.40%) |  |
| High school graduate | 1772 (23.97%) | 367 (22.27%) | 597 (24.02%) | 18 (17.65%) | 643 (24.51%) | 108 (28.12%) |  |
| College degree or above | 3989 (53.96%) | 943 (57.22%) | 1338 (53.84%) | 47 (46.08%) | 1411 (53.79%) | 190 (49.48%) |  |
| **Marital status, n (%)** |  |  |  |  |  |  | 0.002 |
| Married/cohabiting | 4418 (59.77%) | 969 (58.80%) | 1576 (63.42%) | 60 (58.82%) | 1516 (57.80%) | 213 (55.47%) |  |
| Widowed/divorced/separated | 2432 (32.90%) | 569 (34.53%) | 752 (30.26%) | 34 (33.33%) | 902 (34.39%) | 123 (32.03%) |  |
| Never married | 542 (7.33% | 110 (6.67%) | 157 (6.32%) | 8 (7.84%) | 205 (7.82%) | 48 (12.50%) |  |
| **PIR, n (%)** |  |  |  |  |  |  | <0.001 |
| < 1.3 | 1867 (25.26%) | 409 (24.82%) | 582 (23.42%) | 45 (44.12%) | 659 (25.12%) | 118 (30.73%) |  |
| 1.3-3.5 | 3388 (45.83%) | 723 (43.87%) | 1136 (45.71%) | 42 (41.18%) | 1251 (47.69%) | 164 (42.71%) |  |
| > 3.5 | 2137 (28.91%) | 516 (31.31%) | 767 (30.87%) | 15 (14.71%) | 713 (27.18%) | 102 (26.56%) |  |
| **PIR, mean ± SE** | 2.62 ± 1.53 | 2.71 ± 1.57 | 2.71 ± 1.54 | 2.02 ± 1.36 | 2.57 ± 1.51 | 2.42 ± 1.55 | <0.001 |
| **Smoke at least 100 cigarettes in life, n (%)** |  |  |  |  |  |  | <0.001 |
| Yes | 3668 (49.62%) | 782 (47.45%) | 1245 (50.10%) | 61 (59.80%) | 1280 (48.80%) | 201 (52.34%) |  |
| No | 3724 (50.38%) | 866 (52.55%) | 1240 (49.90%) | 41 (40.20%) | 1343 (51.20%) | 183 (47.66%) |  |
| **Hypertension, n (%)** |  |  |  |  |  |  | <0.001 |
| Yes | 4124 (55.79%) | 646 (39.20%) | 1337 (53.80%) | 69 (67.65%) | 1721 (65.61%) | 271 (70.57%) |  |
| No | 3268 (44.21%) | 1002 (60.80%) | 1148 (46.20%) | 33 (32.35%) | 902 (34.39%) | 113 (29.43%) |  |
| **Diabetes, n (%)** |  |  |  |  |  |  | <0.001 |
| Yes | 1682 (22.75%) | 180 (10.92%) | 497 (20.00%) | 74 (29.37%) | 770 (29.36%) | 161 (41.93%) |  |
| No | 5394 (72.97%) | 1409 (85.50%) | 1892 (76.14%) | 173 (68.65%) | 1717 (65.46%) | 203 (52.86%) |  |
| Borderline | 316 (4.27%) | 59 (3.58%) | 96 (3.86%) | 5 (1.98%) | 136 (5.18%) | 20 (5.21%) |  |
| **Day moderate recreational activities, n (%)** |  |  |  |  |  |  | <0.001 |
| <= 3 day | 1579 (21.36%) | 348 (21.12%) | 578 (23.26%) | 37 (14.68%) | 549 (20.93%) | 67 (17.45%) |  |
| >=4 day | 1275 (17.25%) | 361 (21.91%) | 438 (17.63%) | 45 (17.86%) | 381 (14.53%) | 50 (13.02%) |  |
| No reported | 4538 (61.39%) | 939 (56.98%) | 1469 (59.11%) | 170 (67.46%) | 1693 (64.54%) | 267 (69.53%) |  |
| **Self-reported osteoarthritis, n (%)** | 1541 (20.85%) | 268 (16.26%) | 461 (18.55%) | 45 (17.86%) | 665 (25.35%) | 102 (26.56%) | <0.001 |
| **BMI_25_, kg/m2, mean ± SE** | 23.37 ± 4.58 | 20.64 ± 2.11 | 22.40 ± 2.80 | 29.83 ± 5.88 | 23.63 ± 3.18 | 35.28 ± 5.86 | <0.001 |
| **BMI_10prior_, kg/m2, mean ± SE** | 28.82 ± 6.63 | 23.21 ± 2.91 | 27.26 ± 3.73 | 28.91 ± 4.87 | 32.05 ± 5.96 | 40.90 ± 9.63 | <0.001 |
| **BMI_baseline_ kg/m2, mean ± SE** | 29.56 ± 6.74 | 22.22 ± 2.06 | 27.42 ± 1.38 | 24.32 ± 2.79 | 35.11 ± 4.85 | 40.38 ± 8.51 | <0.001 |

Mean ± SD for continuous variables: the P value was calculated by the Weight-adjusted analysis of variance. (%) for categorical variables: the P value

was calculated by the weighted Rao-Scott χ2 test. Abbreviation: BMI, body mass index. PIR, poverty income ratio.
