# Supplementary material for: Weight change patterns across adulthood are associated with the risk of osteoarthritis: a population-based study
Source: Aging Clin Exp Res. 2024 Jun 27;36(1):138. doi: 10.1007/s40520-024-02792-w (PMC11211181; doi:10.1007/s40520-024-02792-w)
Supplement: Supplementary file 4 — Supplementary file4 (DOCX 24 KB) [file 40520_2024_2792_MOESM4_ESM.docx]

| Supplementary 4: Baseline characteristics of study participants in NHANES 2013-2018 based on weight change patterns from age 25 to 10 years before the survey. | | | | | | | |
| --- | --- | --- | --- | --- | --- | --- | --- |
| Characteristics |  | Weight change patterns from age 25 to 10 years before the survey | | | | | P value |
|  | Total | Stable normal weight | Maximum | Obesity to | Non-obesity to obesity | Stable obesity |  |
|  |  |  | overweight | Non-obesity |  |  |  |
| **N** | 7392 | 2082 (28.17%) | 2648 (35.82%) | 143 (1.93%) | 2094 (28.33%) | 425 (5.75%) | <0.001 |
| **Age, years, mean ± SE** | 64.79 ± 9.34 | 63.48 ± 9.35 | 65.47 ± 9.48 | 62.32 ± 8.85 | 66.08 ± 9.07 | 61.49 ± 8.00 | <0.001 |
| **Gender, n (%)** |  |  |  |  |  |  | <0.001 |
| Men | 3661 (49.53%) | 855 (41.07%) | 1469 (55.48%) | 83 (58.04%) | 1034 (49.38%) | 220 (51.76% |  |
| Women | 3731 (50.47%) | 1227 (58.93%) | 1179 (44.52%) | 60 (41.96%) | 1060 (50.62%) | 205 (48.24%) |  |
| **Race/ethnicity, n (%)** |  |  |  |  |  |  | <0.001 |
| Mexican American | 874 (11.82%) | 156 (7.49%) | 314 (11.86%) | 25 (17.48%) | 315 (15.04%) | 64 (15.06%) |  |
| Other Hispanic | 758 (10.25%) | 189 (9.08%) | 308 (11.63%) | 16 (11.19%) | 207 (9.89%) | 38 (8.94%) |  |
| Non-Hispanic White | 3138 (42.45%) | 861 (41.35%) | 1110 (41.92%) | 45 (31.47%) | 954 (45.56%) | 168 (39.53%) |  |
| Non-Hispanic Black | 1632 (22.08%) | 398 (19.12%) | 571 (21.56%) | 40 (27.97%) | 494 (23.59%) | 129 (30.35%) |  |
| Other Race | 990 (13.39%) | 478 (22.96%) | 345 (13.03%) | 17 (11.89%) | 124 (5.92%) | 26 (6.12%) |  |
| **Education level, n (%)** |  |  |  |  |  |  | <0.001 |
| Under high school | 1631 (22.06%) | 420 (20.17%) | 563 (21.26%) | 54 (37.76%) | 497 (23.73%) | 97 (22.82%) |  |
| High school graduate | 1772 (23.97%) | 485 (23.29%) | 647 (24.43%) | 35 (24.48%) | 490 (23.40%) | 115 (27.06%) |  |
| College degree or above | 3989 (53.96%) | 1177 (56.53%) | 1438 (54.31%) | 54 (37.76%) | 1107 (52.87%) | 213 (50.12%) |  |
| **Marital status, n (%)** |  |  |  |  |  |  | 0.002 |
| Married/cohabiting | 4418 (59.77%) | 1215 (58.36%) | 1653 (62.42%) | 90 (62.94%) | 1224 (58.45%) | 236 (55.53%) |  |
| Widowed/divorced/separated | 2432 (32.90%) | 702 (33.72%) | 831 (31.38%) | 39 (27.27%) | 722 (34.48%) | 138 (32.47%) |  |
| Never married | 542 (7.33% | 165 (7.93%) | 164 (6.19%) | 14 (9.79%) | 148 (7.07%) | 51 (12.00%) |  |
| **PIR, n (%)** |  |  |  |  |  |  | <0.001 |
| < 1.3 | 1867 (25.26%) | 542 (26.03%) | 596 (22.51%) | 60 (41.96%) | 536 (25.60%) | 133 (31.29%) |  |
| 1.3-3.5 | 3388 (45.83%) | 902 (43.32%) | 1233 (46.56%) | 53 (37.06%) | 1015 (48.47%) | 185 (43.53%) |  |
| > 3.5 | 2137 (28.91%) | 638 (30.64%) | 819 (30.93%) | 30 (20.98%) | 543 (25.93%) | 107 (25.18%) |  |
| **PIR, mean ± SE** | 2.62 ± 1.53 | 2.69 ± 1.57 | 2.70 ± 1.53 | 2.11 ± 1.49 | 2.54 ± 1.49 | 2.38 ± 1.54 | <0.001 |
| **Smoke at least 100 cigarettes in life, n (%)** |  |  |  |  |  |  | 0.138 |
| Yes | 3668 (49.62%) | 1038 (49.86%) | 1284 (48.49%) | 82 (57.34%) | 1038 (49.57%) | 226 (53.18%) |  |
| No | 3724 (50.38%) | 1044 (50.14%) | 1364 (51.51%) | 61 (42.66%) | 1056 (50.43%) | 199 (46.82%) |  |
| **Hypertension, n (%)** |  |  |  |  |  |  | <0.001 |
| Yes | 4124 (55.79%) | 860 (41.31%) | 1478 (55.82%) | 74 (51.75%) | 1416 (67.62%) | 296 (69.65%) |  |
| No | 3268 (44.21%) | 1222 (58.69%) | 1170 (44.18%) | 69 (48.25%) | 678 (32.38%) | 129 (30.35%) |  |
| **Diabetes, n (%)** |  |  |  |  |  |  | <0.001 |
| Yes | 1682 (22.75%) | 211 (10.13%) | 502 (18.96%) | 34 (23.78%) | 755 (36.06%) | 180 (42.35%) |  |
| No | 5394 (72.97%) | 1804 (86.65%) | 2030 (76.66%) | 106 (74.13%) | 1229 (58.69%) | 225 (52.94%) |  |
| Borderline | 316 (4.27%) | 67 (3.22%) | 116 (4.38%) | 3 (2.10%) | 110 (5.25%) | 20 (4.71%) |  |
| **Day moderate recreational activities, n (%)** |  |  |  |  |  |  | <0.001 |
| <= 3 day | 1579 (21.36%) | 468 (22.48%) | 604 (22.81%) | 19 (13.29%) | 411 (19.63%) | 77 (18.12%) |  |
| >=4 day | 1275 (17.25%) | 419 (20.12%) | 477 (18.01%) | 27 (18.88%) | 298 (14.23%) | 54 (12.71%) |  |
| No reported | 4538 (61.39%) | 1746 (83.86%) | 2117 (79.95%) | 116 (81.12%) | 1556 (74.31%) | 316 (74.35%) |  |
| **Self-reported osteoarthritis, n (%)** | 1541 (20.85%) | 531 (20.05%) | 27 (18.88%) | 538 (25.69%) | 109 (25.65%) | 336 (16.14%) | <0.001 |
| **BMI_25_, kg/m2, mean ± SE** | 23.37 ± 4.58 | 20.49 ± 2.08 | 22.78 ± 2.70 | 30.54 ± 7.07 | 24.12 ± 3.19 | 35.01 ± 5.73 | <0.001 |
| **BMI_10prior_, kg/m2, mean ± SE** | 28.82 ± 6.63 | 22.55 ± 1.87 | 27.33 ± 1.39 | 24.94 ± 2.57 | 34.81 ± 4.99 | 40.66 ± 8.99 | <0.001 |
| **BMI_baseline_ kg/m2, mean ± SE** | 29.56 ± 6.74 | 24.38 ± 4.15 | 28.75 ± 4.16 | 26.85 ± 5.34 | 34.06 ± 6.06 | 38.67 ± 9.35 | <0.001 |

Mean ± SD for continuous variables: the P value was calculated by the Weight-adjusted analysis of variance. (%) for categorical variables: the P value was calculated

by the weighted Rao-Scott χ2 test. Abbreviation: BMI, body mass index. PIR, poverty income ratio.
